# Tea's anti‐obesity properties, cardiometabolic health‐promoting potentials, bioactive compounds, and adverse effects: A review focusing on white and green teas

**DOI:** 10.1002/fsn3.3595

**Published:** 2023-08-15

**Authors:** Behnaz Abiri, Shirin Amini, Mahdi Hejazi, Farhad Hosseinpanah, Afshin Zarghi, Faeze Abbaspour, Majid Valizadeh

**Affiliations:** ^1^ Obesity Research Center, Research Institute for Endocrine Sciences Shahid Beheshti University of Medical Sciences Tehran Iran; ^2^ Department of Nutrition Shoushtar Faculty of Medical Sciences Shoushtar Iran; ^3^ Department of Nutrition, School of Public Health Iran University of Medical Sciences Tehran Iran; ^4^ Department of Pharmaceutical Chemistry, School of Pharmacy Shahid Beheshti University of Medical Sciences Tehran Iran

**Keywords:** anti‐obesity, cardiometabolic diseases, green tea, healthy effects, white tea

## Abstract

Tea is one of the most commonly consumed beverages in the world. Morocco, Japan, and China have consumed green tea for centuries. White tea, which is a variety of green teas, is very popular in China and is highly revered for its taste. Presently, both teas are consumed in other countries around the world, even as functional ingredients, and novel research is constantly being conducted in these areas. We provide an update on the health benefits of white and green teas in this review, based on recent research done to present. After a general introduction, we focused on tea's anti‐obesity and human health‐promoting potential, adverse effects, and new approaches to tea and its bioactive compounds. It has been found that the health benefits of tea are due to its bioactive components, mainly phenolic compounds. Of these, catechins are the most abundant. This beverage (or its extracts) has potential anti‐inflammatory and antioxidant properties, which could contribute to body weight control and the improvement of several chronic diseases. However, some studies have mentioned the possibility of toxic effects; therefore, reducing tea consumption is a good idea, especially during the last trimester of pregnancy. Additionally, new evidence will provide insight into the possible effects of tea on the human gut microbiota, and even on the viruses responsible for SARS‐CoV‐2. A beverage such as this may favor beneficial gut microbes, which may have important implications due to the influence of gut microbiota on human health.

## Graphical abstract

We provide an update on the health benefits of white and green teas in this review, based on recent research done to present. After a general introduction, we have focused on the tea's anti‐obesity and human health‐promoting potentials, adverse effects, and new approaches to the tea and its bioactive compounds. It has been found that tea's health benefits are due to its bioactive components, mainly phenolic compounds. Of these, catechins are most abundant. This beverage (or its extracts) has potential anti‐inflammatory and antioxidant properties, which could contribute to body weight control and improvement of several chronic diseases. However, some studies have mentioned the possibility of toxic effects, so reducing tea consumption is a good idea, especially during pregnancy's last trimester. Additionally, new evidence will provide insight into the possible effects of tea on human gut microbiota and even on the viruses responsible for SARS‐CoV‐2. A beverage like this may favor beneficial gut microbes, which may have important implications due to the influence gut microbiota has on human health.

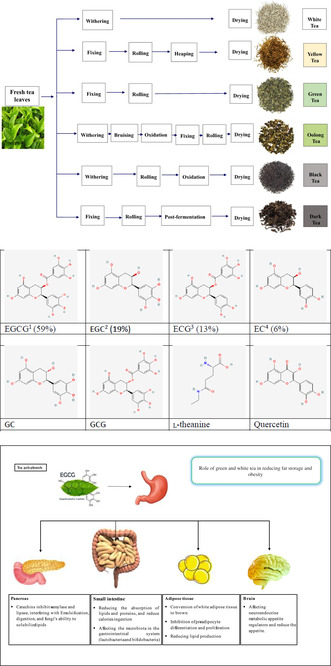

## 1 INTRODUCTION

The extensive use of medicinal herbs in traditional medicine has gained recognition over the years as resources for drug discovery and development. Among the most widely consumed beverages, tea is highly valued for its taste, aroma, and a variety of socio‐cultural reasons (McKinley & Jamieson, 2009); therefore, its health benefits are currently being extensively studied (Chen et al., 2011). The tropical and temperate regions in Asia, South America, and Africa are the main origins of tea plants. This plant belongs to the Theaceae family. Most of the Theaceae family are found in China, Sri Lanka, Japan, and India (Naveed et al., 2018).

It is made by infusing the leaves and shoots of *Camellia sinensis* var. *sinensis* (McKinley & Jamieson, 2009). Tea is classified according to the degree of fermentation, which occurs via enzymatic oxidation of polyphenols by polyphenol oxidases and other oxidative enzymes (Kouhihabibidehkordi et al., 2021). White and green teas are not fermented, and enzyme activity is inactivated via thermal treatment (Kouhihabibidehkordi et al., 2021; Muller et al., 2010). Red tea is semi‐fermented, and black tea is highly fermented (Kouhihabibidehkordi et al., 2021). Therefore, white tea contains the most antioxidants among all types of tea (Kouhihabibidehkordi et al., 2021).

It appears that the antioxidant properties of tea are derived from the presence of flavonoids and polyphenols (Kouhihabibidehkordi et al., 2021). Despite the lack of consensus on this definition, white tea is generally regarded as a type of green tea produced in specific locations, mostly in China's Fujian Province (Muller et al., 2010). Tea leaves that are covered with downy and white hairs are used to make white tea, receiving its name as a result. The first buds and leaves were selected and subjected to minimal processing by simple drying. White tea provides a soft, aromatic flavor and floral and fruit notes in its infusion, which makes its organoleptic qualities highly valued by consumers (Hilal & Engelhardt, 2007; Muller et al., 2010). According to some studies, white tea contains less caffeine and more antioxidant compounds than green tea (Hilal & Engelhardt, 2007; Muller et al., 2010).

Given the similarities between green tea and white tea, and the fact that tea is one of the most consumed beverages in the world, it is constantly being the subject of research. This manuscript provides an overview of tea and highlights its bioactive compounds and their potential health benefits. The focus is on white and green teas, which have potential anti‐obesity properties, to address the global obesity epidemic. Additionally, it acknowledges the need to consider potential adverse effects to promote safe consumption. Overall, this comprehensive review aims to enhance the knowledge of tea's properties, health benefits, and risks while encouraging further research in anti‐obesity and disease prevention areas. This review stands out from others by placing a particular emphasis on white and green tea, allowing for a more detailed examination of their health benefits and risks. It also specifically focuses on the anti‐obesity properties of tea and provides a deeper understanding of its effects and potential therapeutic applications. This distinguishes it from other reviews that may have a narrower scope. Altogether, the review's specific focus and comprehensive approach make it unique among published reviews on tea.

## 2 METHODS

The data were obtained according to the results of the original and review articles associated with white tea, green tea, anti‐obesity, and health. For this purpose, we have searched the literature from the PubMed, Web of Science, EMBASE, and Scopus database with the keywords “white tea,” “green tea,” “*Camellia sinensis*,” “tea,” “anti‐obesity,” “cardiometabolic health,” “health,” “place of origin,” “secondary metabolites,” “quantitative aspects,” “bioavailability,” “pharmacokinetics,” “metabolism,” and “drug interaction.” We then chose relevant free‐access full texts and reviewed the appropriate articles. We considered suitable articles published in English. On the other hand, studies without English language access were excluded. To determine the current position of white and green teas in human health, this review focuses on research published since 2018. Our review incorporates both animal and human studies. In addition, we searched some of the references in the selected articles to better clarify the associated topics.

## 3 TEA VARIETIES

A subtropical or mountainous climate and slightly acidic soils are the best conditions for growing tea in an intensive monoculture (Vishnoi et al., 2018). In general, young shoots are picked, although they vary depending on the tea variety (Vishnoi et al., 2018).

**FIGURE 1:**
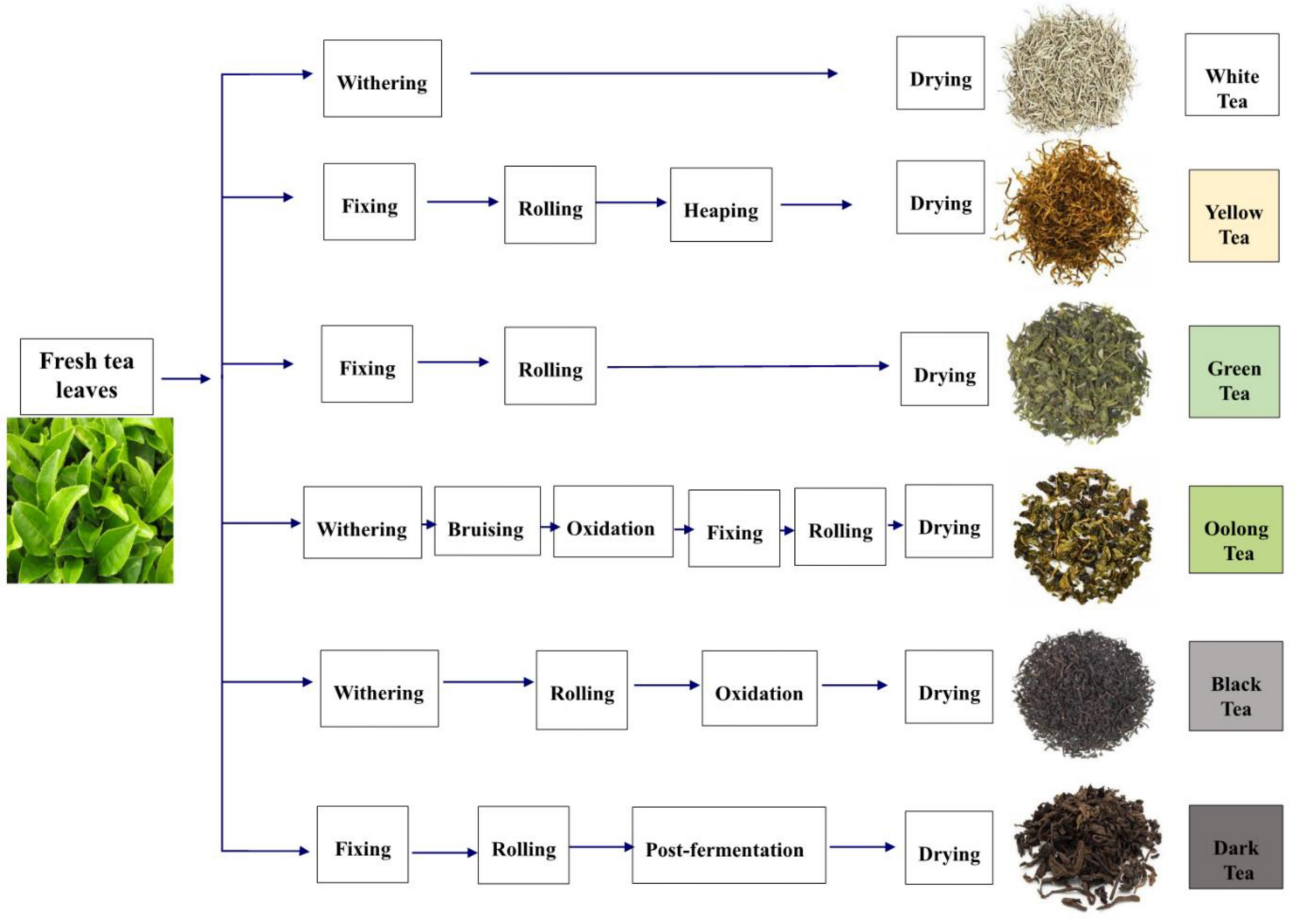
Different tea varieties and processing steps.

Tea leaves are processed in various ways after harvest. Six varieties can be clearly distinguished depending on the process (Figure 1; Wang et al., 2019). There have been thousands of years of processing techniques (Wang et al., 2019). Table 1 summarizes the main processes to which they are subjected. The types of tea differ in their chemical and sensory properties depending on the fermentation process used (Chen et al., 2020; Wang et al., 2019).

**TABLE 1:** Processes involved in tea production.

Production process	Alterations in leaves
Withering	The evaporation of some water as a result of the sun's heat
Fixing	The application of heat to stop oxidation reactions in leaves
Rolling	A method of shaping leaves so they are more manageable
Drying	The final process of removing the remaining moisture
Heaping	The process of stacking leaves (to add some humidity)
Bruising	Breaking the leaves through a process
Oxidation	Degradation of leaf structure and compounds by endogenous enzymes
Post‐fermentation	Process of fermentation caused by exogenous microorganisms on leaves, which modifies its chemical, nutritional, and organoleptic properties

Green, white, yellow, oolong, black, and dark teas are the most common types of tea. Green tea is made by steaming fresh leaves at high temperatures, which inactivates oxidative enzymes while preserving polyphenol content (Fang et al., 2019; Vishnoi et al., 2018). White tea is made from fresh shoots and young leaves and undergoes the least amount of processing, resulting in high levels of antioxidant compounds, despite being oxidized by at least 5% (Chen et al., 2020; Fang et al., 2019; Vishnoi et al., 2018). There was a partial oxidation process in both yellow and oolong tea, with oxidative levels ranging from 10% to 20% and 30% to 60%, respectively (Fang et al., 2019; Vishnoi et al., 2018). The oxidation process occurs between 80% and 95% in black tea, losing some nutrients, but also providing a softer taste (Fang et al., 2019; Vishnoi et al., 2018). Finally, dark tea is treated with exogenous microorganisms after fermentation, whereas the other teas are only exposed to enzyme‐induced oxidation (Fang et al., 2019).

In the final stage of tea production, the tea is dried at various temperatures based on the type of tea used (Pérez‐Burillo et al., 2019). Tea composition changes following drying, particularly for thermolabile molecules (Pérez‐Burillo et al., 2019). Therefore, the antioxidant capacity and phenol content, which are measured using the Folin–Ciocalteu method, are altered during drying (Pérez‐Burillo et al., 2019). In addition, chemical markers have been used to make this step less damaging (Pérez‐Burillo et al., 2019).

## 4 TEA COMPOSITION

**FIGURE 2:**
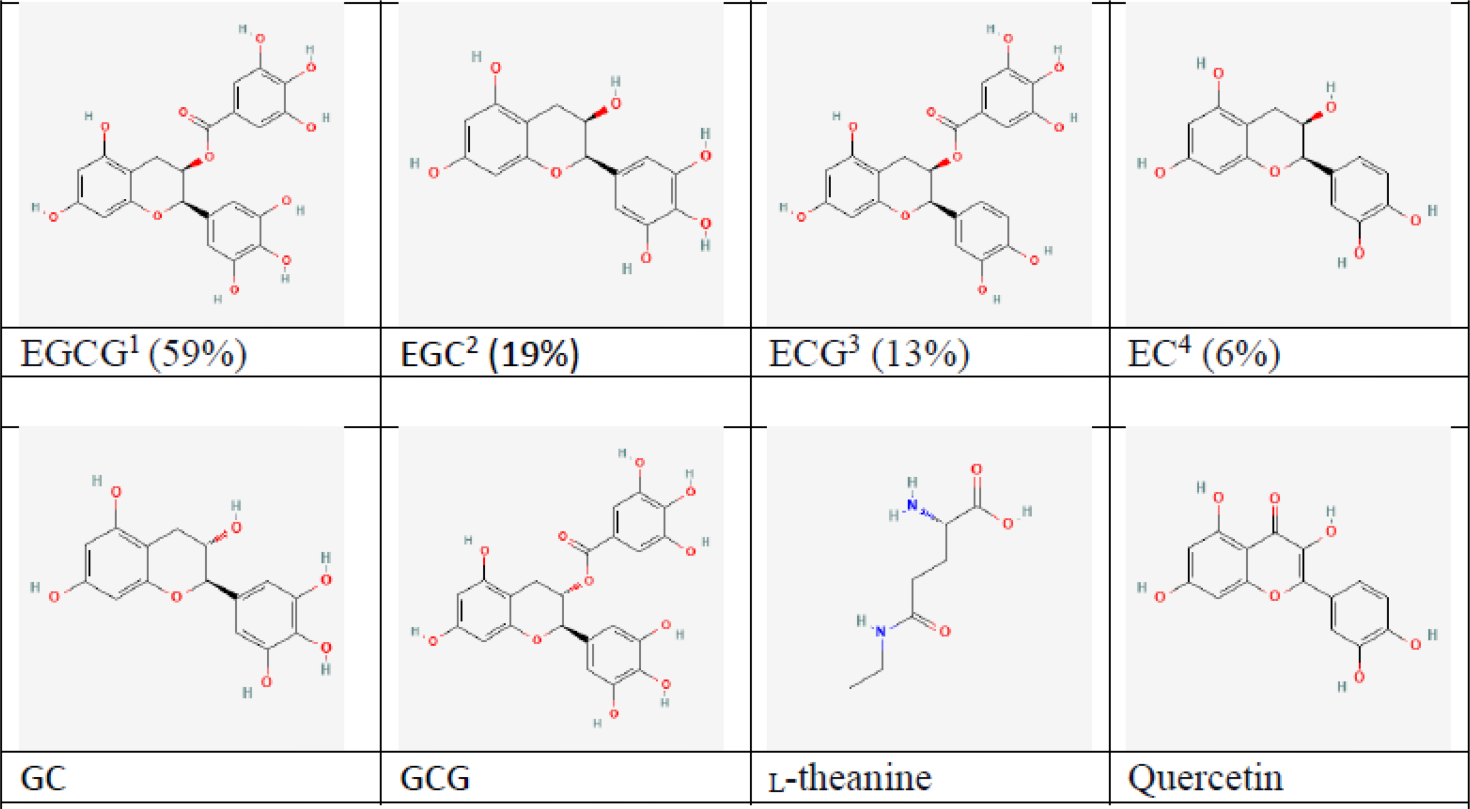
Structure of tea composition. *Source*: ^1^ https://pubchem.ncbi.nlm.nih.gov/compound/65064#section=2D‐Structure; ^2^ https://pubchem.ncbi.nlm.nih.gov/com‐pound/72277; ^3^ https://pubchem.ncbi.nlm.nih.gov/compound/107905#section=2D‐Structure; ^4^ https://puchem.ncbi.nlm.nih.gov/com‐pound/72276.

Tea leaves have a complex composition (Figure 2). In general, tea leaves contain 15%–20% protein (dry weight), with enzymes and amino acids, such as theanine or 5‐N‐ethylglutamine, tryptophan, tyrosine, leucine, or lysine (Hajiaghaalipour et al., 2016; Vishnoi et al., 2018; Wen et al., 2020). Tea leaves also contain vitamins E, C, and B, as well as trace elements (Ca, Zn, and Mg; Peng et al., 2020; Zhang et al., 2019). Moreover, tea composition also includes another class of compounds, such as polyphenols, particularly catechins, methylxanthines, theobromine, caffeine, and theophylline (Hajiaghaalipour et al., 2016; Vishnoi et al., 2018), or aromatic substances (Wen et al., 2020; Zhang et al., 2019). However, heavy metals or pesticides can also be found, which are toxic to humans and should be considered (Zhang et al., 2019).

Despite various methods of processing, catechins, caffeine, and amino acids in fresh tea leaves are the most important determinants of commercial tea quality (Wen et al., 2020). Tea catechins include gallocatechin (GC), epicatechin (EC), epigallocatechin (EGC), epicatechin gallate (ECG), gallocatechin gallate (GCG), and epigallocatechin gallate (EGCG), of which the latter is the most studied (Chen et al., 2017; Wang et al., 2019). Theanine, one of the most abundant amino acids found in tea, has received considerable attention in recent years (Williams et al., 2019). Quercetin is a flavonoid found in various foods and plants, including green tea (Fox et al., 2020).

Polyphenol concentrations in white tea range from 10.60 to 25.95 g per 100 g, while in green tea they range from 13.7 to 24.7 g per 100 g (Hilal & Engelhardt, 2007; Tan et al., 2017). White and green teas had the highest concentrations of catechins, with EGCG accounting for approximately 59%, EGC (19%), ECG (13%), and EC (6%; Cabrera & Gimenez, 2010; Furase et al., 2009; Zhao et al., 2011). It was reported that catechin content in tea ranges from 9.89 to 17.00 g/100 g in green tea, from 7.94 to 16.56 g/100 g in white tea and from 0.74 to 10.00 g/100 g in black tea (Espinosa et al., 2012; Hilal & Engelhardt, 2007). There have also been reports of catechin levels in white tea ranging from 2.76 to 9.34 g/100 g (Tan et al., 2017). As for EGCG, it ranges from 4.40–9.60 g/100 g in green tea to 5.23–9.49 g/100 g in white tea (Cabrera et al., 2003). According to Cabrera et al. (2003), green tea leaves contain more than 80 mg/g EGCG, whereas black tea contains less than 30 mg/g.

Catechins are the major phenolic compounds in tea infusions (Zhao et al., 2019). In vivo, catechins undergo different metabolic transformations, with the liver and gut serving as major metabolic organs (Chen et al., 2017). In the circulation, the metabolites of these compounds are distributed to various organs and tissues, where they exert most of their effects (Chen et al., 2017). As EGCG contains the most phenolic hydroxyl groups, it is the most active antioxidant and has the highest bioactivity potential (Zhao et al., 2019).

## 5 SECONDARY METABOLITES

There are a variety of secondary metabolites in tea, including catechins, amino acids (primarily L‐theanine), caffeine, and aroma compounds that provide it with its unique color, aroma, and flavor, as well as the ability to improve human health (Yang et al., 2021). Most of these compounds are formed during the growing process (preharvest stage) and the processing stage (postharvest stage; Yang et al., 2021). Previous studies have investigated the formation of functional‐ or quality‐associated metabolites in enzymatic and nonenzymatic reactions (Zeng et al., 2019, 2020), and the formation of tea metabolites has been explored from the perspective of plant biology.

## 6 ANALYTICAL METHODS FOR QUANTIFICATION OF SECONDARY METABOLITES

Spectrophotometry is the most commonly used analytical method for determining the total tea polyphenol content. Compared to Iron (II) D‐tartrate, Folin–Ciocalteu chromogenic reagent is more stable and can meet the current quantitative analysis of total tea polyphenols to a certain extent. Since this method oxidizes the ‐OH groups in tea polyphenols, some non‐tea polyphenols can also be easily oxidized, so the theoretical value of the result is higher than the actual value. Although the measurement of total tea polyphenols using spectrophotometry is complicated, rapid detection of these compounds has numerous applications. Total tea polyphenols have been increasingly determined using near‐infrared spectroscopy with nondestructive detection and electrochemical sensors capable of rapid on‐site detection (Sun et al., 2022).

The high‐performance liquid chromatography–UV(/DAD) method is a suitable analytical method for the analysis of individual tea polyphenols, especially catechins. Although mass spectrometry is highly sensitive compared with UV/DAD detectors, tea polyphenols, such as electrospray ionization (ESI) sources, can cause serious matrix effects on ion sources. The UHPLC–MS/MS technique is clearly useful for analyzing low concentrations of tea polyphenols, meeting sensitivity and selectivity requirements for qualitative and quantitative analyses (Sun et al., 2022).

However, during the analysis process, it is still necessary to avoid the problem of matrix effects. In this case, the dilution factor was expanded to compensate for the sacrificing sensitivity. Consequently, the development of specific purification materials, such as metal–organic frameworks and functional graphene materials, for UHPLC–MS/MS analysis of tea polyphenols is of high importance (Sun et al., 2022).

In recent years, high‐resolution mass spectrometry technologies, such as Q‐TOF and Q‐Orbitrap, have become popular for the analysis of tea metabolomics because of their fast acquisition speed, high full scan sensitivity, and high mass resolution. There is extensive evidence that high‐resolution mass spectrometry has proven useful in discovering new tea polyphenols, discriminating between isomers, screening markers, and qualitatively analyzing target and non‐targeted metabolites in complex matrices. Accordingly, derivatization and isotope labeling are more appropriate for tea polyphenol identification (Sun et al., 2022).

## 7 ORAL BIOAVAILABILITY OF METABOLITES PRESENT IN *C. SINENSIS*

Only a small fraction of tea catechins is absorbed in the intestinal tract after drinking tea and is therefore considered bioavailable, reaching the bloodstream or tissues (Cai et al., 2018). Catechin concentrations in green tea infusions range from 3250 to 4410 mg/L, according to a study (Koch et al., 2018). Studies have shown that less than 5% of the tea catechins consumed orally reach the systemic circulation in rats (Catterall et al., 2003; Lin et al., 2007), and 1.68% are present in human plasma (0.16%), urine (1.1%), and feces (0.42%) after tea consumption for over 6 h (Warden et al., 2001). Following oral administration of tea to rats, approximately 14% of epigallocatechin, 31% of epicatechin, and less than 1% of epigallocatechin‐3‐gallate were detected in blood (Chen et al., 1997). For humans, after consuming 3 g of decaffeinated green tea, the maximum plasma concentrations of EGCG, EGC, and EC were 0.57, 1.60, and 0.6 μM, respectively (Yang et al., 1998).

It should be noted that the differences between in vitro and in vivo studies can be attributed to the low bioavailability of green tea catechins. Stability, absorption rate, and efflux affect the bioavailability. The degradation and conjugation of catechins are associated with extreme pH conditions in the stomach, intestinal tract, and related digestive enzymes. As no specific receptors have been found on the surface of small intestinal epithelial cells that are capable of carrying EGCG into cells, catechins are thought to pass through the intestinal epithelium by passive diffusion, including paracellular and transcellular diffusion. As a result, catechin absorption rates are low (Ye & Augustin, 2018).

It is possible to improve catechin bioavailability by using nanostructure‐based drug delivery systems, modifying molecules, and co‐administering catechins with other active ingredients. By encapsulating tea catechins in protein‐, carbohydrate‐, and lipid‐based nanoparticles, we were able to enhance the bioavailability and sustainability of catechin release in the body. Molecular modifications such as peracetylating EGCG protect hydroxyl groups on EGCG from oxidative degradation until it is deacetylated into its parent EGCG by esterases in cells (Lam et al., 2004), reducing the biotransformation and efflux of EGCG. When catechins are administered or formulated in combination with other drugs or bioactive compounds, a synergistic effect occurs, resulting in improved absorption and inhibition of the efflux transporter activity (Cai et al., 2018).

## 8 METABOLISM OF ECGC

There are many health benefits associated with green tea consumption, mainly due to the main bioactive compound, EGCG, which is a flavone‐3‐ol polyphenol present in green tea. Oral administration of EGCG has been reported to be first absorbed in the intestine, but its bioavailability is poor due to its oxidation, metabolism, and efflux (Gan et al., 2018).

The gut microbiota is crucial for EGCG metabolism in the intestine. Studies have indicated that EGCG can be deconjugated and degraded both in vitro and in vivo by gut microbiota. It was reported that the radioactivity in rat blood and most tissues remained low for 4 h following oral administration of isotope‐labeled EGCG and increased after 8 h, achieving the maximum level after 24 h (Kohri, Matsumoto, et al., 2001). This suggests that EGCG is metabolized by gut microbes prior to absorption.

A significant amount of conversion, including hydrolysis, occurs in the gut microbiota to degrade EGCG. Takagaki et al. indicated that EGCG was first hydrolyzed to EGC and gallic acid by rat intestinal bacteria and bacterial strains, including *Raoultella planticola*, *Enterobacter aerogenes*, *Klebsiella pneumoniae*sub species (subsp.) pneumoniae, and *Bifidobacterium longum*subsp. Moreover, this study suggests that rat intestinal microbiota is involved in the metabolism of EGCG. After the hydrolysis of EGCG, the EGC product underwent a series of conversions and degradations, and 5‐(3,5‐dihydroxyphenyl)‐4‐hydroxyvaleric acid was found to be the main metabolite of EGCG in both the cecal contents and feces of rats (Takagaki & Nanjo, 2010). Additionally, it was reported that 5‐(3′,5′‐dihydroxyphenyl)‐γ‐valerolactone was absorbed in the body after degradation of EGCG by intestinal bacteria, and its glucuronide form is the major urinary metabolite of EGCG (Kohri, Matsumoto, et al., 2001; Takagaki & Nanjo, 2010). Furthermore, EGCG is methylated in vivo. Kohri, Nanjo, et al. (2001) discovered 4′,4″‐di‐O‐methyl‐EGCG as the main metabolite of EGCG in rats following intravenous administration, and Lambert et al. (2006) detected 4”‐O‐methyl‐EGCG and 4′,4″‐di‐O‐methyl‐EGCG in mouse small intestine, colon, liver, and prostate following intragastrical administration of EGCG. Based on these results, metabolic pathways of EGCG may differ depending on its administration routine, while gut microbiota‐mediated metabolism appears to be crucial for the absorption of its metabolites when consumed orally.

## 9 PHARMACOKINETICS AND PHARMACOLOGY

Extensive research has been conducted on the pharmacokinetics of green tea catechins in healthy individuals from various perspectives, including the catechin source, dose–response, frequency, and condition of administration (Hu et al., 2018). Generally, catechins in green tea are rapidly absorbed after ingestion and reach their peak plasma concentrations within 1–5 h of ingestion with a one‐peak plasma concentration versus time course followed by a multiphasic decrease consisting of a distribution phase and an elimination phase (Law et al., 2017). The main (60%–90%) form of EGCG and ECG in plasma is in the free form, whereas EGC and EC are mostly in their glucuronide, methylate, and sulfate conjugates. After consumption, most catechins are eliminated from the plasma within 24 h, and they have half‐lives of 2–10 h (Hu et al., 2018).

As measured by plasma *C*_max_ or AUC values, the internal exposure to free EGCG in human clinical trials ranges from 0.0035 to 7.36 μM for *C*_max_, and 0.06 to 24.93 μM × h for AUC0‐24 h following a single oral dose of 72.8–1200 mg EGCG (equivalent to 1.2–20 mg EGCG/kg for a 60‐kg person) (Sun et al., 2009). After repeated oral dosing, plasma *C*_max_ values of free EGCG were reported to be between 0.3 and 0.63 μM, and the mean AUC0‐24 h of 2.0–5.8 μM × h following 800 mg EGCG/day from Polyphenon E taken with a meal for 4 weeks (Hu et al., 2018); and could reach up to 6.10 μM for plasma *C*_max_ and 19.7 μM × h for plasma AUC0‐t following 800 mg EGCG/day under fasted states for 10 days. Under several different dosing conditions, plasma‐free EGCG concentrations in healthy human subjects were more similar to those observed in rats and dogs fed a 13‐week diet (Isbrucker et al., 2006), and these values were at least two orders of magnitude lower than those found in dogs that had fasted (Kapetanovic et al., 2009).

Green tea contains catechin, caffeine, theanine, polysaccharides, and other chemicals that possess pharmacological activities. These compounds possess antioxidant, antitumor, hypoglycemic, and other health benefits. Tea polyphenols are widely used as natural antioxidants in the food and cosmetic industries. Furthermore, catechins in green tea also have important effects in the prevention and treatment of diabetes, cancer, hepatitis, microbial/viral infections, and skin inflammation (Zhao et al., 2022).

## 10 THE BENEFITS OF TEA AND TEA‐RELATED BIOACTIVE COMPOUNDS

Strong evidence supports the health benefits of tea in treating obesity and other diseases (Zhao et al., 2019). The highest concentration of bioactive compounds is found in green and white teas, so they also have the greatest health benefits (Cheng, 2019; Pérez‐Burillo et al., 2018). The general consensus is that moderate tea consumption (3–5 cups per day, 250 mL per cup) can achieve these effects (Cheng, 2019; Pérez‐Burillo et al., 2018). Thus, this review focuses on studies dealing with the health properties of white and green teas, their extracts, and their main bioactive compounds.

### 10.1 Influences on lipolysis and obesity

It is well known that obesity is a chronic multifactorial disorder described by an excessive fat accumulation and that it is an increasing medical problem (Sultane Halima & Cambaza, 2020). It increases the risk of several diseases including diabetes, pulmonary diseases, cancers, and osteoarthritis (Sultane Halima & Cambaza, 2020). According to the World Health Organization (WHO), in 2016, more than 1.9 billion adults (>18 years old) were overweight, with over 650 million were obese, 39% of men and 40% of women were overweight and 13% of the global population (11% of men and 15% of women) were obese (Sultane Halima & Cambaza, 2020).

Despite several interventions to combat obesity, including promotion of healthy living and lifestyle habits, medications, surgical procedures, physical activity, and low‐calorie diets, results have not been satisfactory and their effects do not last long, and sometimes patients suffer adverse effects as a result of such interventions (Diepvens et al., 2007; Rodgers et al., 2012; Sultane Halima & Cambaza, 2020). Therefore, there is a need to explore effective, convenient, tolerable, and economical alternatives. In recent years, natural phytotherapeutic supplements have become increasingly popular, and tea is the most widely used (Auvichayapat et al., 2008; Dostal et al., 2016; Sultane Halima & Cambaza, 2020).

Various kinds of teas and their bioactive compounds have been shown to combat obesity from anthropometric and biochemical perspectives (Abe & Inoue, 2020; Bagheri, Rashidlamir, Ashtary‐Larky, Wong, Alipour, et al., 2020; Hodges et al., 2020; Jiménez‐Zamora et al., 2016; Kobayashi & Ikeda, 2017; Lee et al., 2019; Lin, Shi, et al., 2020; Luo et al., 2020; Rocha et al., 2016; Sultane & Cambaza, 2020; Xu, Yang, Li, et al., 2020; Yonekura et al., 2020). Researchers have shown that the most important effects of these compounds have been in reducing lipid absorption and increasing fat oxidation, which reduces triglycerides, cholesterol, and leptin and increases energy expenditure in both in vitro and animal studies (Abe & Inoue, 2020; Jiménez‐Zamora et al., 2016; Lee et al., 2019; Luo et al., 2020; Rocha et al., 2016; Xu, Yang, Li, et al., 2020). The main changes that have been identified in epidemiological studies include a decrease in body weight, a change in body mass index (BMI), a lower waist/hip ratio (WHR), and a lower visceral and body fat percentage (Abe & Inoue, 2020; Bagheri, Rashidlamir, Ashtary‐Larky, Wong, Alipour, et al., 2020; Jiménez‐Zamora et al., 2016; Kobayashi & Ikeda, 2017; Lin, Shi, et al., 2020; Sultane & Cambaza, 2020; Yonekura et al., 2020).

In particular, white tea extract improves lipid metabolism, which helps modulate metabolic syndrome (Lee et al., 2019). By reducing the levels of low‐density lipoproteins in the blood, these extracts reduced the levels of apolipoprotein B, cholesterol, and triglycerides. As a result, triglyceride accumulation inside cells is also significantly reduced (Bagheri, Rashidlamir, Ashtary‐Larky, Wong, Alipour, et al., 2020). Triglycerides are a major component of adipose tissue and are crucial for insulin sensitivity and energy expenditure (Liu et al., 2019).

Among the tea varieties studied for its anti‐obesogenic properties, green tea is the most studied (Abe & Inoue, 2020; Jiménez‐Zamora et al., 2016; Kobayashi & Ikeda, 2017; Lin, Shi, et al., 2020; Rocha et al., 2016). Since the effectiveness of the intervention depends on the dose and type of extract, as well as the duration of the intervention, there is a dose–response relationship (Jiménez‐Zamora et al., 2016; Kobayashi & Ikeda, 2017). EGCG has been shown to reduce the body weight of rats by up to 29% when injected intraperitoneally (Kao et al., 2000). It has been suggested that this effect is caused by molecular inhibition of fatty acid synthase by EGCG (Kao et al., 2000). Furthermore, catechins inhibit α‐amylase and lipase, interfering with emulsification, digestion, and the ability of fungi to solubilize lipids (Sultane Halima & Cambaza, 2020).

Green tea consumption was found to be inversely related to obesity in 232 middle‐aged Japanese women (Yonekura et al., 2020). In a study by Lin, Shi, et al. (2020), daily intake of <500 mg of green tea for 12 weeks resulted in improved body weight and BMI, specifically for periods longer than 12 weeks at a dose of <800 mg per day. Evidence has shown that individuals who consumed 400 mL of tea daily for 10 years had a lower percentage of body fat and a smaller waist/hip ratio (Jiménez‐Zamora et al., 2016; Zhao et al., 2015). Despite its protective effects against hyperlipidemia, high‐density lipoproteins were not affected, but they did influence low‐density lipoproteins and triglyceride levels (Jiménez‐Zamora et al., 2016; Rocha et al., 2016). The induction of antioxidant responses in adipose tissue has been described as another mechanism that induces oxidative stress (Jiménez‐Zamora et al., 2016). Moreover, tea interferes with the formation of micelles in the intestine, thereby inhibiting lipid absorption (Rocha et al., 2016).

Polyphenols in tea appear to contribute significantly to its anti‐obesogenic effects. In some studies, consumption of green tea polyphenols caused a reduction in the amount of adipose tissue lipogenesis and activated AMPK, causing a reduction in plasma lipid levels as well as stimulating fatty acid oxidation in obese rats (Jiménez‐Zamora et al., 2016; Luo et al., 2020). According to a study conducted on Japanese women, coffee and chlorogenic acid resulted in a reduction in BMI because of their synergistic effects (Yonekura et al., 2020).

When consumed at doses >300 mg per day, caffeine in green tea plays a role in weight loss (Abe & Inoue, 2020) by altering thermogenesis and the metabolism of white adipose tissue. The effects of green tea catechins on plasma lipid homeostasis have been demonstrated in vitro and in animal experiments (Jiménez‐Zamora et al., 2016; Rocha et al., 2016). They reduce plasma cholesterol, low‐density lipoprotein, and triglycerides (Jiménez‐Zamora et al., 2016; Rocha et al., 2016). Catechins inhibit the synthesis of fatty acids and lowered β‐oxidation (Jiménez‐Zamora et al., 2016). EGCG increased the expression of acetyl coenzyme A carboxylase‐1 in rats and reduced fatty tissue accumulation (Jiménez‐Zamora et al., 2016). EGCG may also reduce endothelial dysfunction caused by oxidized low‐density lipoproteins via the Jagged‐1/Notch pathway (Rocha et al., 2016). Fecal excretion in animals is increased by green tea catechins, which reduce cholesterol levels (Xu, Yang, Li, et al., 2020). By altering the emulsification process, catechins also reduce digestive lipase, gastric lipase, and pancreatic lipase activities, thus reducing fat digestion and absorption (Jiménez‐Zamora et al., 2016). Additionally, tea catechins contribute to an increase in bile acid synthesis by inhibiting the reabsorption of bile acids from the small intestine (Jiménez‐Zamora et al., 2016). Bile acids (BAs) are powerful metabolic regulators that can facilitate fat elimination and subsequently reduce diet‐induced obesity owing to their specific function in fat metabolism (Sun et al., 2019; Troup et al., 2015; Zhao et al., 2015). A significant reduction in diet‐induced obesity was associated with increased plasma BAs levels, which led to an increase in whole‐body energy expenditure and heat dissipation (Liaset et al., 2011). White tea extracts derived from EGCG and ECG were found to affect the genes involved in cholesterol metabolism, according to Luo et al. (Luo et al., 2020). Increased mRNA levels of thermogenic genes, including Ucp1 and Ucp2, were induced by EGCG in mice (Jiménez‐Zamora et al., 2016). In addition, leptin levels are significantly reduced in rats treated with EGCG (Jiménez‐Zamora et al., 2016). A 12‐week study conducted on healthy Japanese men found that consumption of 690 mg of tea catechins significantly reduced body weight and fat mass (Nagao et al., 2005). The results of a meta‐analysis demonstrated a direct correlation between catechin concentration and reduced BMI, body weight, and waist‐to‐hip ratio (Yonekura et al., 2020). Although in vitro studies have demonstrated clear anti‐obesogenic effects, epidemiological studies have revealed high heterogeneity.

In addition to being short in duration and small in sample size, racial and ethnic differences, and initial BMI, make it difficult to establish definitive links between tea consumption and its effects on obesity (Abe & Inoue, 2020; Kobayashi & Ikeda, 2017; Rocha et al., 2016). Hence, tea consumption should be combined with a healthy diet and adequate exercise (Abe & Inoue, 2020; Kobayashi & Ikeda, 2017; Lin, Shi, et al., 2020; Yonekura et al., 2020). Researchers have conducted studies to investigate the effects of tea consumption and its relationship to physical activity (Bagheri, Rashidlamir, Ashtary‐Larky, Wong, Alipour, et al., 2020). Bagheri, Rashidlamir, Ashtary‐Larky, Wong, Alipour, et al. (2020) reported that green tea extracts improved exercise‐induced body composition by increasing weight, BMI, waist‐to‐hip ratio, and body fat percentage. The intervention consisted of an 8‐week training program with three training sessions per week and a green tea supplement of 500 mg daily. Adiponectin concentrations, body composition, and inflammation improved in this study (Bagheri, Rashidlamir, Ashtary‐Larky, Wong, Alipour, et al., 2020). According to these results, a combination of daily tea consumption and regular exercise programs can provide great benefits to obese patients. Although green tea has been shown to have anti‐obesity effects, these findings are contradictory and inconclusive. Additionally, the optimal intake is yet to be determined (Jurgens et al., 2012). Moreover, existing evidence suggests that epigallocatechin and caffeine from tea leaves exert independent but synergistic effects on weight loss (Sirotkin & Kolesárová, 2021).

**FIGURE 3:**
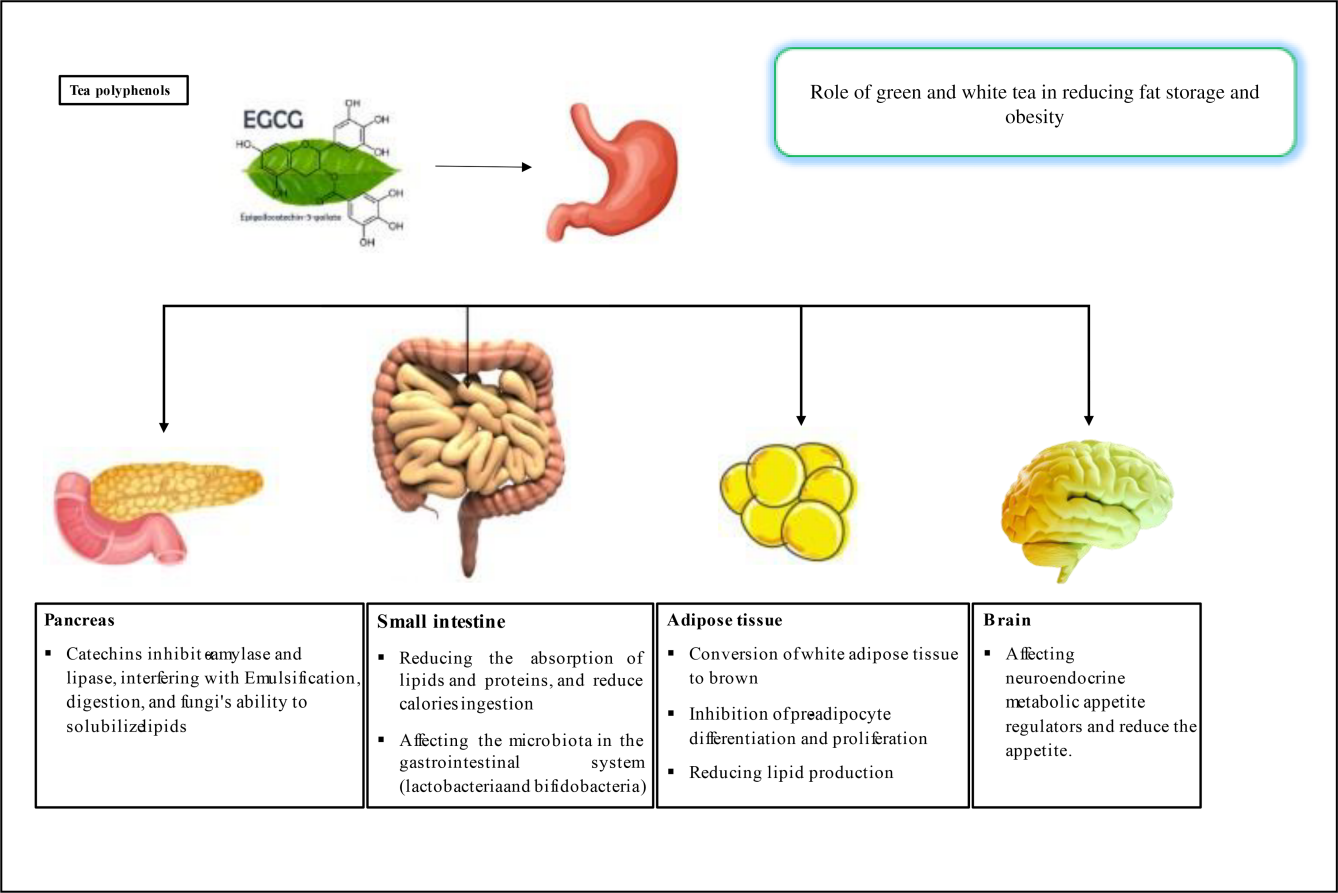
Mechanisms of body weight reduction by green and white teas.

In summary, several pathways may be involved in the reduction in fat storage by tea molecules (Figure 3; Cao, Zhao, Xiao‐Yu, et al., 2019; Huang et al., 2014; Pan et al., 2016; Rothenberg et al., 2018; Shang et al., 2021; Silvester et al., 2019; Willems et al., 2018; Xu et al., 2021; Yang et al., 2016):

- They affect neuroendocrine metabolic appetite regulators and reduce the appetite (Huang et al., 2014);
- They reduce calorie intake by reducing the absorption of lipids and proteins (Pan et al., 2016; Shang et al., 2021);
- They affect the microbiota of the gastrointestinal system (lactobacteria and bifidobacteria), which are responsible for digestion (Rothenberg et al., 2018; Yang et al., 2016);
- They inhibit preadipocyte differentiation and proliferation (Pan et al., 2016);
- They enhance lipolysis and lipid metabolism (Pan et al., 2016; Yang et al., 2016);
- They diminish lipid production (Huang et al., 2014; Yang et al., 2016);
- They oxidize white adipose tissue, stimulate its conversion to brown adipose tissue, burn it, and increase calories expended through heat production (Huang et al., 2014; Silvester et al., 2019; Willems et al., 2018);
- They help modulate the gut microbiota and excrete fecal lipids (Cao, Zhao, Xiao‐Yu, et al., 2019; Huang et al., 2014).

However, it is important to consider that the benefits of green tea and its components are only apparent when large amounts are consumed. The recommendation of a research group was 100–460 mg of epigallocatechin gallate with 80–300 mg of caffeine administered over the course of at least 12 weeks (Vázquez Cisneros et al., 2017). Another recommended drinking 3–4 cups of strong tea (600–900 mg catechins) every day for at least 8 weeks (Yang et al., 2018).

### 10.2 Effects on lipid profile, hypertension, and cardiovascular diseases

Cardiovascular diseases (CVD) include various pathologies that affect the circulatory system. Several animal studies have demonstrated the cardioprotective effects of tea, including antioxidant, anti‐inflammatory, antihypertensive, anti‐thrombogenic, and lipid‐lowering properties (Babu et al., 2008; Cao, Zhao, Gan, et al., 2019; Zhang et al., 2015).

Having an energy metabolism disorder seriously affects a person's life. An abnormally high lipid level contributes considerably to the development of cardiovascular diseases through hypercholesterolemia, a modifiable risk factor. White tea also plays an important role in the regulation of lipid metabolism (Luo et al., 2020). Despite a lack of clinical studies, animal studies have confirmed the health benefits of white tea. Islam (2011) reported that rats treated with white tea extract showed an effective decrease in lipid‐related parameters. White tea extract significantly decreased total cholesterol and low‐density lipoprotein‐cholesterol (LDL‐c). In addition to animal tests, in vitro studies have been conducted to identify the hypolipidemic effects of white tea. Researchers found strong lipolytic and antiadipogenic effects of white tea extract on human subcutaneous preadipocytes (Söhle et al., 2009). White tea extract treatment inhibits the expression of transcription factors involved in adipogenesis, such as CCAAT‐enhancer‐binding protein α (C/EBPα), adipocyte determination and differentiation factor 1 (ADD1), and sterol regulatory element‐binding protein 1‐c (SREBP1‐c; Söhle et al., 2009). Tenore et al. (2013) found that white tea extract increased the low‐density lipoprotein receptor (LDLR) binding activity and reduced lipase activity . At the protein level, apolipoprotein A1 (ApoA1), the major component of high‐density lipoproteins (HDLs), has been shown to have increased expression (Tenore et al., 2013). Recent studies have shown that white tea extracts have hypolipidemic properties. The direct mechanism of white tea extract in lipid regulation remains an open question because of the lack of a systematic analysis.

Several transcription factors have been identified to regulate LDLR expression. LDLR is regulated by SREBP2 (Sterol Regulation element‐binding Protein 2; Luo et al., 2020), and peroxisome proliferator‐activated receptor δ (PPARδ) has recently been shown to function in the LDLR promoter (Luo et al., 2020).

In vitro studies have demonstrated that white tea improves abnormal lipid metabolism. While most people are able to tolerate first‐line anti‐cholesterolemic drugs such as statins, they are ineffective in treating familial hypercholesterolemia because they can aggravate joint and muscle pain (Luo et al., 2020). White tea is widely known to have health benefits for humans because it contains high concentrations of natural antioxidants as a result of its minimal processing. Novel insights into lipid homeostasis mediated by white tea extracts were revealed in vitro. Overall, white tea extract can be considered a potential hypolipidemic beverage.

Observational studies have shown that green tea consumption is negatively correlated with the risk of cardiovascular diseases (Rocha et al., 2016). A study conducted in Tokyo, Japan assessed the diet and coronary angiography results of 612 participants. A significant inverse association was observed between increased consumption of green tea and coronary artery disease prevalence. A lower prevalence of the disease was observed in those who consumed more than three cups per day than in those who consumed <1 day. In addition, higher fruit and vegetable consumption is associated with this low prevalence (Bagheri, Rashidlamir, Ashtary‐Larky, Wong, Grubbs, et al., 2020). According to a meta‐analysis by Xu et al., the consumption of green tea has been found to significantly lower systolic and diastolic blood pressure. The authors suggested that decreasing oxidative stress and reducing vascular inflammation could ameliorate the endothelial function (Xu, Yang, Ding, & Chen, 2020).

The effects of tea on CVD may be mediated by various compounds (Hodges et al., 2020). By enhancing the production of nitric oxide in the body, green tea catechins can inhibit inflammatory cytokines and platelet aggregation and improve endothelial function and blood flow (Xu, Yang, Ding, & Chen, 2020). Studies have shown that EGCG is a potent antihypertensive (Hodges et al., 2020; Kishimoto et al., 2020) and capable of vasodilating blood vessels in a dose‐dependent manner. Furthermore, research has shown that endurance exercise enhances the effects of EGCG, although further studies are required to confirm this claim (Xu, Yang, Ding, & Chen, 2020). Caffeine, also, affects blood vessel homeostasis (Hodges et al., 2020; Kishimoto et al., 2020). In animals and humans, theanine and quercetin lower blood pressure, which reduces the risk of CVD. The antioxidant effects of Quercetin also reduce oxidative stress and improve vascular function by affecting the renin‐angiotensin‐aldosterone system (Fox et al., 2020). Although epidemiological studies have shown slight associations, there are still many questions that must be answered (Bagheri, Rashidlamir, Ashtary‐Larky, Wong, Grubbs, et al., 2020; Kishimoto et al., 2020).

### 10.3 Effects on glycemic control and diabetes

In diabetes mellitus, excessive blood glucose levels are caused by insulin resistance and inadequate insulin release (Yan et al., 2020). Bioactive compounds from green tea and white tea have been studied extensively both in vitro and in animal studies (Atia et al., 2020; Fox et al., 2020; Sanlier et al., 2018; Yan et al., 2020). In a study, it was found that white tea consumption improved glucose tolerance and blood glucose levels in mice. In addition, β‐cells in the islets of Langerhans were found to possess antioxidant and anti‐inflammatory effects (Xia et al., 2020). Researchers have found increased insulin sensitivity after 2 months of drinking white tea in pre‐diabetic rats (Fox et al., 2020). Therefore, controlling blood glucose levels and reducing diabetes risk are benefits of white tea consumption (Atia et al., 2020; Fox et al., 2020; Shang et al., 2021).

In peripheral tissues such as skeletal muscles and adipocytes, green tea is believed to lower blood glucose levels through altered insulin resistance (Yan et al., 2020). Treatment with green tea polyphenols for 15 days reduces glucose levels in diabetic mice by 44% (Yan et al., 2020). An eight‐week study using Wistar rats treated with green tea was also conducted. Inhibiting inflammatory factors and reducing ROS levels reduces diabetic complications despite poor hypoglycemic effects (Sanlier et al., 2018). It has also been proven that EGCG supplements can boost glucose tolerance, prevent Langerhans islet destruction, modulate gene expression of certain enzymes, and contribute to insulin sensitivity (Yan et al., 2020) and low serum levels of glucose (Fox et al., 2020). In contrast, tea flavonoids can inhibit intestinal glucose absorption by suppressing α‐glucosidase activity and increasing glucose transporter 4 (GLUT4), which allows glucose to be absorbed and increases insulin sensitivity (Sanlier et al., 2018). Furthermore, genistein and quercetin in green tea inhibited the activities of the insulin receptor tyrosine kinases IRS‐1 and IRS‐2 (Jiménez‐Zamora et al., 2016). Theaflavins and catechins also reduced hyperglycemia in rats by inhibiting β‐cell damage and increasing insulin activity in in vitro studies (Fox et al., 2020; Yan et al., 2020).

Several epidemiological studies have shown that green tea reduces insulin resistance and ameliorates blood glucose levels. However, this effect was not adequately supported by evidence (Hodges et al., 2020; Liu et al., 2018; Xia et al., 2020; Yu et al., 2017). There was a significant reduction in fasting insulin and blood sugar levels with green tea supplementation in short‐term trials (Liu et al., 2018). Yu et al. (2017) conducted a systematic review of the effects of green tea and green tea extracts on insulin resistance and glycemic control. Participants reported that the extracts were better than drinking due to taste issues, which made the extracts more convenient. Green tea consumption was associated with a decreased risk of type 2 diabetes in a large cohort of Chinese men and women (Yu et al., 2017).

Nevertheless, a study conducted in Shanghai (China) found that people who drank green tea and oolong tea were more likely to develop type 2 diabetes. It was reported that tea drinkers frequently smoked and drank alcohol more often, exercised less, and consumed more calories (Yu et al., 2017). According to these findings, long‐term exploration of tea supplements and their bioactive compounds in humans is warranted to determine whether supplemental tea can reduce diabetes and glycemia.

### 10.4 Anti‐inflammatory activity

Various pathologies and disorders are associated with inflammation and immune system damage. Several inflammatory markers such as C‐reactive protein have been shown to be significantly reduced by drinking green tea (Saeed, Abd El‐Hac, et al., 2017). Additionally, green tea components, such as catechins, have been shown to elevate the production of anti‐inflammatory cytokines including IL‐10, regulating also the synthesis and signaling of IL‐6 and reducing pro‐inflammatory cytokines (such as IL‐1β and TNF‐α; Asbaghi et al., 2019; Cao, Zhao, Gan, et al., 2019; Farzaei et al., 2019; Haghighatdoost & Hariri, 2019; Jiménez‐Zamora et al., 2016; Rahman et al., 2018; Saeed, Abd El‐Hac, et al., 2017; Shang et al., 2021).

In a meta‐analysis (Asbaghi et al., 2019), no significant impact was found on C‐reactive protein. However, TNF‐α levels were significantly reduced. The researchers attributed these results to the low bioavailability of tea bioactive compounds after consumption. Additionally, the authors pointed out that the anti‐inflammatory effects of tea were more evident in cases of severe inflammation (Asbaghi et al., 2019). The polyphenols in tea suppress the expression of iNOS in the liver (Yuan et al., 2006), while green tea decreased the levels of IL‐6 in patients with Polycystic Ovary Syndrome (Ghafurniyan et al., 2014). The results of another study comparing metabolic syndrome patients with healthy people receiving a daily green tea supplement (1 g; 890 mg total catechins) for 28 days demonstrated that the mRNA expression levels of dependent inflammatory genes decreased (Farzaei et al., 2019). Metabolic syndrome can be reversed by green tea catechins, which inhibit inflammatory responses. However, the authors emphasize the need for long‐term interventions (Farzaei et al., 2019).

When diseases like Crohn's disease or inflammatory bowel disease affect the intestinal epithelium, inflammation of the barrier will occur. Green tea extract contains catechins, which improve the intestinal barrier integrity in rat models. Thus, endotoxins derived from the intestine are modulated in their translocation, and the resulting pro‐inflammatory effects are modulated (Farzaei et al., 2019; Haghighatdoost & Hariri, 2019; Rahman et al., 2018). In addition to decreasing the severity of colitis symptoms, EGCG treatment significantly enhanced antioxidant capacity (Farzaei et al., 2019; Haghighatdoost & Hariri, 2019; Rahman et al., 2018). Furthermore, green tea extract decreased the entry of immunoreactive nitrotyrosine into the colon, as well as the overexpression of intercellular adhesion molecule 1 (ICAM‐1; Haghighatdoost & Hariri, 2019). In summary, a variety of chronic inflammatory disorders have been shown to be protected by tea polyphenols (Farzaei et al., 2019; Haghighatdoost & Hariri, 2019; Rahman et al., 2018).

### 10.5 Antioxidant capacity

In general, antioxidants have the ability to substantially combat reactive oxygen species (ROS), which include oxygen‐derived free radicals such as nitric oxide, superoxide anions, and hydroxyl radicals that cause degenerative diseases. Antioxidants are present in a wide variety of plants, but their presence in tea is highly beneficial. The bioactive compounds in many types of tea and herbs protect body tissues from oxidative stress caused by oxygen‐derived free radicals and byproducts of lipid peroxidation (Ryan & Petit, 2010). ROS cause oxidative damage in many diseases (Chen et al., 2017; Jiménez‐Zamora et al., 2016; Saeed, Naveed, et al., 2017). Many tea compounds, in particular polyphenols, are high in antioxidants (Chen et al., 2017; Jiménez‐Zamora et al., 2016; Saeed, Naveed, et al., 2017; Shang et al., 2021). Thus, tea phenolic compounds have been found to exhibit several different antioxidant pathways (Saeed, Naveed, et al., 2017). Polyphenols react with ROS because of their chemical structure, neutralizing them and preventing their accumulation in organisms (Chen et al., 2017; Saeed, Naveed, et al., 2017). Some compounds, including tea catechins, have been shown to modulate the expression and activity of antioxidant enzymes such as superoxide dismutase and glutathione S‐transferase (Chen et al., 2017; Saeed, Naveed, et al., 2017). Experimental evidence suggests that catechins can continuously elevate enzyme activity and eliminate ROS in animals (Saeed, Naveed, et al., 2017). Furthermore, compounds containing phenols can modulate the activity of certain metabolic pathways and hence diminish the production of oxygen‐free radicals (Chen et al., 2017; Jiménez‐Zamora et al., 2016; Saeed, Naveed, et al., 2017). Chelation inhibits the formation of some oxidation catalysts, as in the case of some minerals such as calcium or iron (Saeed, Naveed, et al., 2017). Tea catechins can also decrease free radical levels in lipids and stimulate β‐oxidation (Jiménez‐Zamora et al., 2016; Saeed, Naveed, et al., 2017). To increase serum antioxidant capacity, tea polyphenols work synergistically with certain vitamins in rodents (Saeed, Naveed, et al., 2017).

An additional bioactive compound found in tea has demonstrated antioxidant effects in the prevention of ROS‐induced damage (Shang et al., 2021; Yan et al., 2020). In addition, theanine reduces toxicity because of pharmacologically induced lipid peroxidation and modulates drug‐induced glutathione peroxidase activity (Yan et al., 2020). A study in animals found that green tea helped the body detoxify xenobiotics by increasing glutathione S‐transferase levels (Yan et al., 2020). There is promising evidence that green tea and its extracts decrease toxin‐induced oxidative stress and can even improve detoxification (Chen et al., 2017). Several xenobiotic compounds, including tobacco smoke, mycotoxins, and heavy metals, are protected by the antioxidant capacity of green tea phenolic compounds (Chen et al., 2017).

The antioxidant activity of tea and its bioactive compounds have been attributed to several mechanisms. A variety of mechanisms are critical for fighting different diseases.

## 11 OTHER BENEFICIAL EFFECTS

Bile acids regulate metabolism in a powerful manner. Female rats were administered different types of teas for 28 days as part of a study conducted by Sun et al. (2019). The metabolism of different bile acids varies according to metabolic studies. Tea may have beneficial health effects by altering bile acid metabolism (Sun et al., 2019). Research has shown that green tea extracts can reverse liver damage caused by toxic contaminants in rats (Yan et al., 2020). Extracts (specifically those rich in EGCG) from green tea have also been shown to ameliorate non‐alcoholic fatty liver disease in animal studies (Sun et al., 2019). The presence of green tea decreases nuclear factor‐kappa β activity, a factor involved in liver inflammation (Khoo et al., 2020). The antioxidant effects of white tea prevent liver damage (Yan et al., 2020). Researchers found that EGCG and white tea extract decreased oxidative damage caused by Benzo (a) pyrene in hepatocytes and red blood cells (Sanlier et al., 2018). Consumption of green tea and EGCG may reduce the levels of liver enzymes and modulate bilirubin levels in humans. Healthy individuals were not affected by this effect, but subjects with hepatic damage were affected (Khoo et al., 2020). Additional studies are needed because of the discrepant results and the possibility of toxic effects.

Different types of tea have demonstrated beneficial effects on bone health in multiple studies (Filippini et al., 2020; Lin, Kan, et al., 2020; Tomaszewska et al., 2018; Yan et al., 2020). In a study by Tomaszewska et al., regular tea drinkers had a reduced risk of hip fractures (Tomaszewska et al., 2018). In the same study, postmenopausal women who drank tea had a higher bone mineral density (Filippini et al., 2020). The effects of green tea have been predominantly described. However, white tea has the greatest protective effects on bone tissues and cartilage (Filippini et al., 2020), while black tea promotes bone mineralization (Tomaszewska et al., 2018). However, the polyphenols in green tea have been reported to enhance bone formation and decrease bone loss (Filippini et al., 2020; Yan et al., 2020), specifically tannic acid (Filippini et al., 2020). EGCG has also been shown in another research to help mitigate bone loss and ameliorate bone microstructure in rats when consumed daily for 3 months (Yan et al., 2020). Many constituents of green tea have estrogenic effects, including catechins, theaflavins, kaempferol, and phenolic acids (Lin, Kan, et al., 2020). In rats exposed to prolonged levels of lead (Pb) or cadmium (Cd), drinking white, black, or green tea is reported to protect bone metabolism and increase bone resorption (Filippini et al., 2020; Yan et al., 2020). There is some evidence that tea consumption can promote bone health; however, further studies are required to prove this hypothesis in vivo (Tomaszewska et al., 2018).

In vitro studies have shown that different types of tea positively affect crystal morphology, density, and size, with green tea showing the best results (Xu, Bai, Yang, & Chen, 2020). There is strong evidence that regular tea consumption leads to a reduced risk of kidney stone occurrence by 13% in women and 22% in men (Nasrul & Sehgal, 2020). This study suggests a dose–response relationship between regular consumption of tea and a reduced risk of kidney stones (Nasrul & Sehgal, 2020). Despite the fact that green tea contains oxalate, a potential crystal‐forming factor, a rat model revealed that supplementing drinking water with green tea could diminish the accumulation of crystals inside the kidneys (Shu et al., 2019). Several mechanisms may be responsible for the reduced risk of lithiasis, including increased fluid intake, diuretic and natriuretic effects, and antioxidant and anti‐inflammatory effects of polyphenols (Nasrul & Sehgal, 2020; Xu, Bai, Yang, & Chen, 2020). In a clinical trial, EGCG from green tea was shown to improve albuminuria in diabetic nephropathy. Activation of diacylglycerol kinase α (DGKα) is the major mechanism that protects kidneys (Kanlaya & Thongboonkerd, 2019). Additionally, EGCG has been demonstrated to have iron‐chelating properties, decreasing ROS production, and preventing kidney damage (Shu et al., 2019). There is still a need for more clinical trials to establish conclusive evidence that supplementation protects the kidneys (Shu et al., 2019).

Many studies have been conducted recently regarding tea's anti‐cancer properties (Ghafurniyan et al., 2014; Gianfredi et al., 1886; Hayashi et al., 2020; Hodges et al., 2020; Wang, Zhao, et al., 2020; Yuan et al., 2006). The different properties of polyphenols in tea contribute to their effectiveness in preventing and controlling cancer progression (Hayashi et al., 2020; Yuan et al., 2006). Several mechanisms have been studied, including antioxidant activity, cell cycle regulation, immune modulation, and epigenetic regulation (Ghafurniyan et al., 2014; Wang, Zhao, et al., 2020).

Overall, the polyphenols in tea have antitumor properties (Hayashi et al., 2020; Yuan et al., 2006), promoting apoptosis (Hayashi et al., 2020; Hodges et al., 2020), and chemopreventive properties (Yuan et al., 2006). In vitro studies have shown that tea catechins inhibit cancer cell growth through several mechanisms (Ghafurniyan et al., 2014; Wang, Zhao, et al., 2020). ECGC alters epigenetic signals in cancer cells by altering histone modification and DNA methylation (Wang, Zhao, et al., 2020). Furthermore, the same researchers noted that polyphenols from green tea could induce apoptosis and inhibit the growth of cancer cells, in part due to their pro‐oxidant properties (Wang, Zhao, et al., 2020). Green tea consumption is inversely related to some cancers, including lung, oral, and ovarian cancers, with an estimated 19% risk reduction (Hodges et al., 2020). Contradictory results have been reported for other types of cancers (Hodges et al., 2020). Currently, research is focusing mainly on the impact of tea on colorectal cancer (Ghafurniyan et al., 2014; Hayashi et al., 2020; Yuan et al., 2006), breast cancer (Gianfredi et al., 1886; Shirakami & Shimizu, 2018), and prostate cancer (Rogovskii et al., 2019). A meta‐analysis of 142 studies concluded that the link between drinking green tea and reduction of cancer risk was inconclusive (Filippini et al., 2020). Moreover, the majority of studies included were in Asian countries, which are already known for their high levels of tea consumption; therefore, they could not be extrapolated to populations of other regions (Gianfredi et al., 1886). Generally, there are promising in vitro effects for tea extracts at high concentrations, as well as for some individual bioactive constituents (such as EGCE). Despite this, there is no conclusive evidence linking green or white tea consumption to cancer risk (Gianfredi et al., 1886; Hodges et al., 2020; Shirakami & Shimizu, 2018). It would be prudent to conduct more studies on different types of populations and better‐designed studies to confirm these positive findings.

There is growing evidence of the beneficial effects of tea on mental health (Nasrul & Sehgal, 2020), memory and learning (Rahman et al., 2018), anxiety (Williams et al., 2019; Xing et al., 2019; Yan et al., 2020), stress (Williams et al., 2019; Yan et al., 2020), depression (Xing et al., 2019), and neurodegenerative diseases (Khalatbary & Khademi, 2020; Rahman et al., 2018). There are many amino acids in tea, of which theanine is one of the most abundant. It resembles the excitatory neurotransmitter glutamic acid (Glu) and inhibitory neurotransmitter gamma‐aminobutyric acid (GABA; Khalatbary & Khademi, 2020). Hence, theanine has neuroprotective (Khalatbary & Khademi, 2020; Williams et al., 2019; Yan et al., 2020) and stress‐relieving properties during resting (Williams et al., 2019; Yan et al., 2020). Those who consume green tea feel calm and relaxed, making it an anxiolytic (Williams et al., 2019; Yan et al., 2020). Green tea extract enriched with EGCG has been found to be a therapeutic agent for neurodegeneration (Rahman et al., 2018). EGCG has been found to protect against neural injury and Huntington's disease, as well as amyotrophic lateral sclerosis and other neurodegenerative diseases more commonly seen today (Unno et al., 2020). An animal model of Parkinson's disease has shown that green tea extract has neuroprotective and memory‐enhancing properties. In addition to inhibiting amyloid plaque formation, they slow oxidative damage and immunochemical changes (Rahman et al., 2018). The effects of gallic acid and pyrogallol were also apparent, although their potencies were lower than that of EGCG (Rahman et al., 2018). Therapies for Alzheimer's disease target acetylcholinesterase and butyrylcholinesterase. In previous studies, EGCG was the only compound that exhibited significant competitive inhibition of acetylcholinesterase and therefore was capable of increasing acetylcholine concentrations in synaptic terminals (Kakutani et al., 2019). Antioxidant activity and reduced inflammation in the brain were responsible for these results. Dementia is often associated with inflammation of the brain. EGCG improves endothelial function and is neuroprotective (Yoneda et al., 2019). However, not all studies support these findings; Kakutani et al. reported that green tea consumption was not related to ameliorated memory impairment, in contrast to a German cohort study. These findings are attributed to the different metabolic responses resulting from different ethnic and genetic backgrounds (Kakutani et al., 2019).

Many studies have suggested that tea and some of its bioactive compounds, such as EGCG and EGC, have antiviral (Fox et al., 2020; Yan et al., 2020), antimicrobial (Yan et al., 2020), fungicidal (Fox et al., 2020; Kiyama, 2020), and antiparasitic activities (Kiyama, 2020). In the future, the effect of tea on SARS‐CoV‐2 will be an important trend; however, more studies are required to obtain a definitive answer. In a study, three green tea polyphenols (EGCG, ECG, and GCG) were shown to be potential inhibitors of SARS‐CoV‐2 Mpro (Ghosh et al., 2020). As a result, it can fight the coronavirus disease 2019 (COVID‐19) because it is a fundamental enzyme in the process of viral replication and transcription (Reygaert, 2018).

## 12 THE NEGATIVE AND TOXIC IMPACTS OF TEA AND ITS BIOACTIVE COMPOUNDS

Although tea consumption has many health benefits, some adverse effects have also been reported. There have been reports of some tea products being toxic, but mostly at high doses or throughout certain physiological periods (Bedrood et al., 2018; Dekant et al., 2017; EFSA Panel on Food Additives and Nutrient Sources added to Food (ANS) et al., 2018; Hahn et al., 2017; Hu et al., 2018; Jiménez‐Zamora et al., 2016; Kiss et al., 2019; Kobayashi & Ikeda, 2017; Kumar et al., 2020; Lu et al., 2017; Pedra et al., 2019; Rahman et al., 2018; Saeed, Abd El‐Hac, et al., 2017; Shah et al., 2020). In vitro animal models and human studies have evaluated the safety and toxicological effects of tea and its bioactive compounds (Dekant et al., 2017). A number of factors determine the toxicity of a substance, including its concentration, type, and duration of exposure (Bedrood et al., 2018). With the exception of Asian countries, where almost five cups of tea are consumed each day, the rest of the world's countries consume low levels of tea and tea extract (Dekant et al., 2017; EFSA Panel on Food Additives and Nutrient Sources added to Food (ANS) et al., 2018). Most studies, reviews, and metaanalyses mention liver toxicity as one of the most significant complications (Bedrood et al., 2018; Dekant et al., 2017; Hu et al., 2018; Rahman et al., 2018; Saeed, Abd El‐Hac, et al., 2017). The most hepatotoxic component of catechins is EGCG, particularly at high concentrations (Hu et al., 2018; Rahman et al., 2018). Acute toxicity has been demonstrated to be dose‐dependent (Bedrood et al., 2018; Hu et al., 2018). These compounds exhibit pro‐oxidant properties (because of their high concentrations), which are responsible for cytotoxicity in the liver (Bedrood et al., 2018; Saeed, Abd El‐Hac, et al., 2017). EGCG has the ability to induce reactive oxygen species in the mitochondria at high concentrations (Bedrood et al., 2018), which results in oxidative damage that destroys cellular DNA (Hu et al., 2018). Damage caused by elevated lipid peroxidation has also been previously reported (Hu et al., 2018).

According to the European Food Safety Authority, green tea drinkers consume between 90 and 300 mg of EGCG per day from their tea infusions, with a maximum consumption of up to 866 mg per day (EFSA Panel on Food Additives and Nutrient Sources added to Food (ANS) et al., 2018). As a result of the rare incidence of adverse effects occurring in only a few consumers, liver toxicity cannot be established for the regular consumption of traditional tea infusions, although these studies should be taken into consideration (Dekant et al., 2017; Hu et al., 2018; EFSA Panel on Food Additives and Nutrient Sources added to Food (ANS) et al., 2018).

Studies have shown that green tea consumption can lead to nutrient–nutrient interactions (Yonekura et al., 2020). The majority of studies indicate that the bioavailability of folic acid (Bedrood et al., 2018) and iron (Bedrood et al., 2018; Kobayashi & Ikeda, 2017) has declined. In Pakistan, a cross‐sectional study of about 400 pregnant women evaluated the association between tea consumption by pregnant women and the risk of iron deficiency anemia. Serum iron and ferritin levels were higher in non‐tea drinkers, whereas total iron‐binding capacity was higher in tea drinkers. Women who drank tea during pregnancy developed anemia. It is recommended to reduce tea consumption during pregnancy and never drink tea near meals (Shah et al., 2020). Furthermore, serum folate levels decrease during pregnancy (Bedrood et al., 2018). A variety of mechanisms contribute to this effect, including caffeine and other methylxanthines, which may complex nutrients or irritate the gastrointestinal tract (Bedrood et al., 2018; Hu et al., 2018).

Localized gastric effects of subacute toxicity have also been reported (Bedrood et al., 2018). Depending on the dose, there is a spectrum of effects on the gastrointestinal tract, from mild gastric erosion to occasional diarrhea and vomiting (589, 65, 140) to ulceration, dilation, or even severe harm (Hu et al., 2018).

Additionally, tea extracts affect the levels of drug absorption and bioavailability as well as their activity and elimination (Bedrood et al., 2018; Kiss et al., 2019). Green tea tannins decreased the absorption of iron and atropine. Iron, theophylline, aminophylline, warfarin, codeine, atropine, ephedrine, pseudoephedrine, and diphenoxylate/codeine were decreased by tannins in green tea (Bedrood et al., 2018). Evidence suggests that catechins found in green tea modulate P‐gp‐mediated transport (Schonthal, 2011). Through this effect, cancer chemotherapy could be enhanced by increasing intracellular drug concentrations in cancerous cells; however, it might increase the cytotoxicity of normal cells, which may have harmful effects on them (Bedrood et al., 2018).

Animal studies demonstrated that EGCG by inhibition of hepatic CYP3A family and intestinal P‐gp increases the area under the curve (AUC) of diltiazem (Li & Choi, 2008), nicardipin (Choi & Burm, 2009), and verapamil (Chung et al., 2009). In addition, EGCG increased the AUC of irinotecan and its active metabolite, SN38, in animal plasma by inhibiting P‐gp‐mediated biliary excretion (Lin et al., 2008).

Because of the importance of intestinal UGT1A1, an important enzyme for the inactivation of SN‐38, its interactions with green tea should be considered in the initial metabolism of raloxifene and ezetimibe (Mohamed et al., 2010).

An interaction between EGCG, boronic acid‐based proteasome inhibitors, and sunitinib (Ge et al., 2011) has been reported, where EGCG blocks the impact of these chemotherapeutics. When green tea is consumed, sulindac and/or tamoxifen are more effective and have fewer adverse effects.

Other adverse effects of high doses of green tea include headaches, insomnia, and tachycardia (Jiménez‐Zamora et al., 2016; Kobayashi & Ikeda, 2017). It can be found that liver and gastrointestinal toxicity contributed to these toxicological findings (Hu et al., 2018). There is insufficient evidence of thyroid toxicity (Hu et al., 2018). Theanine consumption has not been linked to adverse health effects in humans (Bedrood et al., 2018). The intake of catechins has shown similar results (Bedrood et al., 2018). However, during breastfeeding, high tea consumption can cause sleep disturbances in babies (Bedrood et al., 2018).

The consumption of tea during pregnancy may increase the chance of premature birth as well as different parameters of fetal growth, although the evidence is inconclusive. Recent evidence suggests that consumption of polyphenols by pregnant women, especially during the third trimester, could contribute to fetal ductal constriction, and hence, to a greater risk of low birth weight or other complications (Hahn et al., 2017; Pedra et al., 2019). During the third trimester of pregnancy, anti‐inflammatory and antioxidant effects are counterproductive (Hahn et al., 2017). The reversal of fetal ductal constriction was induced by suspension of flavonoid‐rich drinks (Hahn et al., 2017). Brazilian fetal cardiology guidelines recommend that pregnant women consume fewer products rich in polyphenols, such as tea, during the last trimester of pregnancy (Pedra et al., 2019).

As a result, mild and various side effects have been observed during acute, subacute, and chronic tests of tea consumption and its components. Hepatotoxicity, interactions with drugs and nutrients, gastrointestinal disturbances (such as vomiting and diarrhea), and health consequences in babies are the most common side effects. Green tea infusions are generally safe and rarely cause health problems, and their influence is minimal and rare (Hu et al., 2018; EFSA Panel on Food Additives and Nutrient Sources added to Food (ANS) et al., 2018). In individuals with kidney or liver disease, and during pregnancy or lactation, intake should be restricted to reduce possible side effects (Bedrood et al., 2018).

## 13 INNOVATIVE APPROACHES AND DEVELOPMENTS

Numerous products are currently being tested to benefit from the properties of tea and its biological components (Godočiková, 2019; Peng et al., 2020). Consumers are becoming increasingly interested in green tea supplements. Over the past few years, tea consumption has shifted from tea beverages to tea in solid form. Tea‐based products have been developed, including green tea cake, green tea bread, green tea noodles, or tea ice cream (Peng et al., 2020). They are also referred to as functional foods. By adding tea extracts to white chocolates, the antioxidant activity was positively influenced by more than doubling the amount of polyphenols, flavonoids, and phenolic acids. The best results were obtained when green tea was added (Godočiková, 2019). Using extracts of green tea in the manufacture of yogurt, wheat dough, and biscuits has also shown promising results (Lorenzo & Munekata, 2016).

Certain compounds, such as theanine and polyphenols (specifically green tea catechins), are currently being used as food additives with technological effects, with antioxidants and antibacterial properties as their main characteristics (Lorenzo & Munekata, 2016; Purnamayanti et al., 2020). According to Lorenzo et al., green tea extracts in meat products inhibit the growth of microorganisms and increase the shelf life (Lorenzo & Munekata, 2016). The addition of green tea polyphenols to lamb sausages effectively inhibits both bacterial growth and lipid oxidation (Purnamayanti et al., 2020). Green tea extracts have been found to improve meat quality characteristics such as color and shelf life, and reduce lipid oxidation (Lorenzo & Munekata, 2016; Purnamayanti et al., 2020). However, high‐pressure extraction yields more bioactive ingredients. Therefore, cold processing of white tea may be a viable alternative to hot tea, as a greater amount of its bioactive compounds is retained (Uzuner & Evrendilek, 2019). Green tea can also be used in cosmetic products (Peng et al., 2020) and animal feeds (Wang, Jia, et al., 2020). When green tea extract was added to the diet of laying hens, the eggs produced were of higher quality, free amino acids, fatty acids, and antioxidants (Wang, Jia, et al., 2020).

## 14 CONCLUSION

Among the most consumed beverages worldwide, tea is one of the most widely studied. Tea is potentially advantageous for the prevention and treatment of obesity and various health states associated with oxidative stress, inflammation, obesity, and cancer. A variety of bioactive molecules, primarily phenolic compounds and catechins, have been linked to the health benefits of tea. Most of the beneficial effects of these compounds arise from their antioxidant capacity once they enter systemic circulation. However, in vitro and animal model studies have shown promising results. Even though it may be weak, tea may cause specific toxicity or alter intestinal absorption of some nutrients under certain circumstances. On the other hand, recent findings on modulating the gut microbiota and the new trends in using it as a feed, additive, or material are noteworthy as well. Despite this promising outlook, extensive clinical trials are still required to validate the health benefits of tea. Therefore, a considerable amount of work remains to be conducted.

## AUTHOR CONTRIBUTIONS

**Behnaz Abiri:** Conceptualization (equal); writing – original draft (equal). **Shirin Amini:** Writing – original draft (equal). **Mahdi Hejazi:** Methodology (equal). **Farhad Hosseinpanah:** Writing – review and editing (equal). **Afshin Zarghi:** Conceptualization (equal). **Faeze Abbaspour:** Data curation (equal); writing – original draft (equal). **Majid Valizadeh:** Conceptualization (equal); supervision (equal); writing – review and editing (equal).

## CONFLICT OF INTEREST STATEMENT

None.

## Data Availability

The data that support the findings of this study are available on request from the corresponding author.
